# MR-derived right ventricular parameters can predict pulmonary hypertension

**DOI:** 10.1186/1532-429X-13-S1-P48

**Published:** 2011-02-02

**Authors:** Kayleen Fabini, Amir H Davarpanah, Marie Wasielewski, Jeremy Collins, Timothy J Carroll, Sanjiv J Shah, James C Carr

**Affiliations:** 1 Northwestern University, Chicago, IL, USA

## Introduction

Right heart catheterization (RHC) is required for definitive diagnosis of pulmonary hypertension (PH) and serial echocardiograms or RHC are necessary for long term disease management. However, RHC is invasive, costly and risky for the patient[1]. Echocardiography is a non-invasive alternative but recent studies have questioned its accuracy and reproducibility in the setting of PH[2]. Cardiac magnetic resonance (CMR) has been shown to be helpful in distinguishing PH patients from healthy subjects through visualization of morphologic features such as right atrial and ventricular enlargement, tricuspid regurgitation and abnormal interventricular septal motion[3]. CMR has the additional advantage of producing quantitative measures of right ventricular function, which may have added value in assessing PH.

## Purpose

To evaluate the use of Cardiac MR for measuring right ventricular parameters in order to assess Pulmonary Hypertension.

## Methods

23 patients with PH and 12 controls underwent CMR on a 1.5T Siemens Avanto scanner. All patients underwent RHC to diagnose PH within 1 month of CMR study. The CMR protocol included cine SSFP of the whole heart and phase contrast MRI of the pulmonary valve (PV). The following conventional right ventricular (RV) functional parameters were calculated: ejection fraction (EF), end diastolic volume (EDV) and end systolic volume (ESV). Time to peak systole (TPS), an indirect measure of RV strain, was derived from the volume-time curves of the RV. PV flow analysis was performed and the following parameters were calculated: peak velocity, full-width at half maximum (FWHM), acceleration time (AT) and ejection time (ET).

## Results

**Figure 1:**
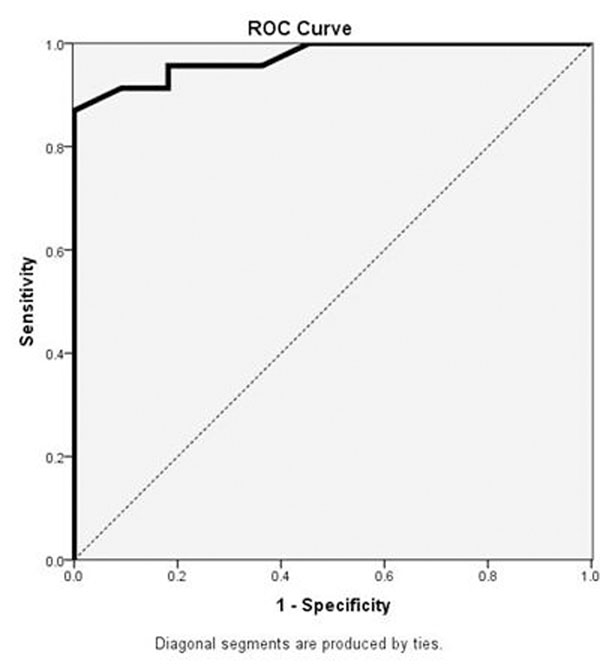
Receiver Operating Characteristic (ROC) curve

The results of RV functional parameters and PV flow values are shown in Tables 1 and 2. TPS was significantly longer for PH patients (p<0.01). The shape of the PV flow curve designated by FWHM was also longer in patients (p<0.05). TPS demonstrated a significant, although relatively weak, correlation with mPAP (r=0.425, p<0.05) and PCWP (r=0.435, p<0.05) measurements on RHC. Calculation of the Area Under Curve for TPS gave a value of 0.972 (p<0.001) (Figure 1). A cut off value of 42.5% for TPS had 91.3% sensitivity and 90.9% specificity for diagnosis of PH.

**Table 1:** Cine MR-derived right ventricular parameters in pulmonary hypertension patients and controls

	Controls	Patients	p-value
RV EF (ml)	46.93±7.91	37.95±14.07	<0.05
EV EDV (ml)	197.50±49.73	192.80±65.04	0.83
RV ESV (ml)	106.39±35.94	127.80±50.83	0.21
RV SV (ml)	91.12±21.01	69.63±32.49	<0.05
TPS (%)	37±4	51±7	<0.01

**Table 2:** Phase contrast MR-derived pulmonary flow parameters in pulmonary hypertension patients and controls

	Controls	Patients	p-value
FWHM (%)	35±6	45±7	<0.05
Velocitymax(cm/s)	67.5±3.3	75.3±23	0.54
AT (%)	17±2	19±3	0.63
ET (%)	46±6	48±3	0.41
AT/ET	0.37±0.03	0.41±0.07	0.15

## Conclusions

TPS, calculated by CMR, may be a useful additional quantitative measure for diagnosis of PH and may be of value in long-term non-invasive assessment of treatment efficacy.
